# Preferences of Adolescents and Young Adults With Epilepsy and Caregivers on Reproductive Health Counseling by Neurologists: A Concept Mapping Study

**DOI:** 10.1016/j.pediatrneurol.2025.08.002

**Published:** 2025-08-11

**Authors:** Laura Kirkpatrick, Erin Friel, Jasmin Rivero-Guerra, Amy Tao, Janani Kassiri, Marie Clements, Christina Briscoe, Page B. Pennell, Jessica G. Burke, Sara Baumann, Traci M. Kazmerski

**Affiliations:** a Department of Pediatrics, University of Pittsburgh School of Medicine, Pittsburgh, Pennsylvania; b Princeton University, Princeton, New Jersey; c Stollery Children’s Hospital/University of Alberta, Edmonton, Alberta, Canada; d Norton Children’s Hospital/University of Louisville, Louisville, Kentucky; e Boston Children’s Hospital/Harvard Medical School, Boston, Massachusetts; f Department of Neurology, University of Pittsburgh School of Medicine, Pittsburgh, Pennsylvania; g University of Pittsburgh School of Public Health, Pittsburgh, Pennsylvania

**Keywords:** Concept Mapping, Epilepsy, Reproductive health, Adolescent health, Counseling

## Abstract

**Objective:**

To ascertain reproductive health counseling priorities of adolescent and young adult women with epilepsy (AWWE) and caregivers during neurology visits.

**Methods:**

We recruited AWWE aged 14-26 years and caregivers from institutional neurology clinics, a research registry, and epilepsy listservs for a Concept Mapping study. Participants (1) brainstormed topics important for counseling of AWWE about reproductive health, (2) sorted topics into categories and rated their importance (on a five-point Likert scale) for AWWE aged 14-17 and 18-26 years, and (3) met to interpret study findings. We included a small subset of the participants in the interpretation meeting to allow meaningful discussion.

**Results:**

Thirty-four AWWE and 20 caregivers generated 37 topics, which were sorted/rated by 35 AWWE and 23 caregivers; seven AWWE and nine caregivers attended the interpretation meeting. Consensus categories included “Hormonal Changes,” “Contraception,” “Sex & Epilepsy,” “Preparing for Pregnancy,” “Pregnancy with Epilepsy,” and “Parenthood & Epilepsy.” For ages 14-17 years, categories rated at least 4.00 for importance included “Hormonal Changes,” “Contraception,” “Sex and Epilepsy,” and “Preparing for Pregnancy.” For ages 18-26 years, all categories were rated at least 4.00. In the interpretation meeting, participants proposed a previsit checklist tool to indicate topics of interest.

**Conclusions:**

AWWE want counseling about reproductive health from neurologists that is tailored by age and more comprehensive than current American Academy of Neurology recommendations. Use of a previsit checklist tool may help identify individual patient and family counseling priorities.

## Introduction

Adolescent and young adult women with epilepsy (AWWE) face distinct risks related to their reproductive health. These risks include the potential of many antiseizure medications (ASMs) to cause congenital malformations and adverse neuro-developmental effects in children exposed *in utero* as well as bidirectional drug interactions between contraceptives and ASMs that may increase the chance of unintended pregnancy or worsen seizure control.^1–4^ These risks are exacerbated by the rate of unplanned pregnancy experienced by adolescents and young adults in the United States— including those with epilepsy—-which remains high compared with other high-income countries.^5–7^ Consequently, AWWE would likely benefit from education and counseling about reproductive health and epilepsy including from their neurologists.

In 2017, the American Academy of Neurology (AAN) published the Women with Epilepsy quality measure, which recommends that all neurologists (including pediatric providers) counsel women with epilepsy who are at least 12 years old at least annually about at least two of three topics: folic acid supplementation, interactions between ASMs and contraception, and effects of ASMs on pregnancy and/or fetal/child development.^8^ However, this quality measure was developed without input from AWWE or caregivers (i.e., parents/household members).^8^ It is unclear if its content reflects the priorities of AWWE, particularly those who are nulliparous and not actively considering pregnancy, as well as their caregivers. The priorities of these groups may differ from those of women with epilepsy who are considering or have experienced pregnancy.

In this study, we used Concept Mapping to ascertain priority topics for reproductive health counseling in the neurology context for AWWE and caregivers.^9,10^ Concept Mapping includes (1) Brainstorming, (2) Sorting and Rating, and (3) an Interpretation Meeting.^9,10^ During brainstorming, participants generate ideas in response to an open-ended prompt. During sorting, participants view the responses to brainstorming and sort those responses into conceptually related categories. During rating, participants rate each response from brainstorming on a quantitative scale (for example, a five-point Likert scale). During the interpretation meeting, the study team facilitates a discussion with participants to elicit their interpretations of the results. The groups of participants do not need to be identical for each stage. We chose to use Concept Mapping methodology because it is a participatory mixed method for exploring opinions in a group and delineating consensus, making it suitable for integrating patient/caregiver perspectives in best practice development.^9–12^

## Methods

**FIGURE 1.**
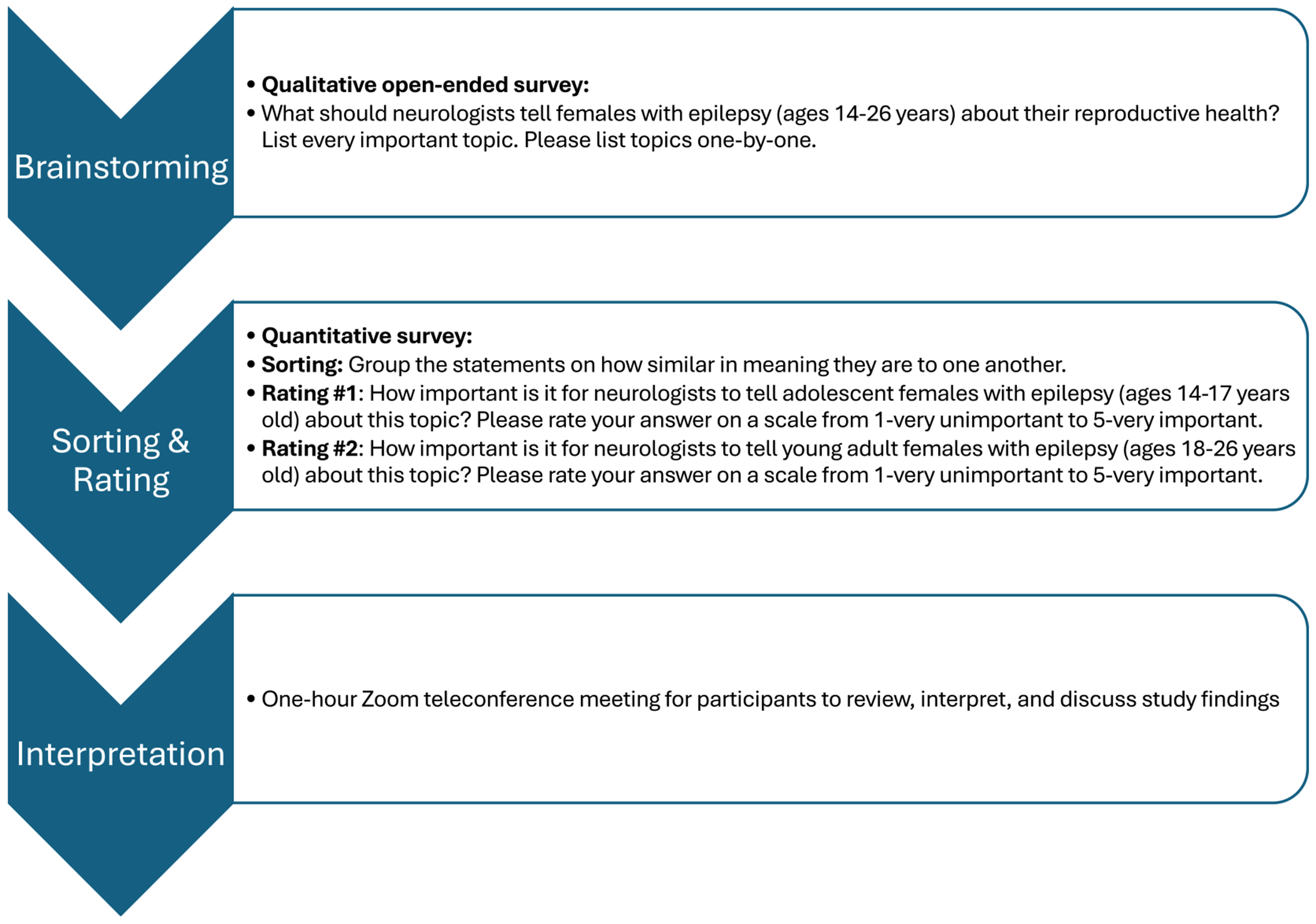
Concept Mapping methods. The color version of this figure is available in the online edition.

Stages of Concept Mapping as applied to this study are diagrammed in Fig 1. We conducted this study using Group-wisdom software (Ithaca, NY, USA).

### Sample size

As a qualitative method, sample size in Concept Mapping is based on achieving data saturation.^9,10,13,14^ The literature on Concept Mapping indicates that 15 participants per stage is sufficient to appropriately conduct Concept Mapping analyses.^13^ Consequently, we set a minimum goal to recruit at least 15 AWWE and at least 15 caregivers for brainstorming, sorting, and rating. For the interpretation meeting, to keep the discussion manageable, we planned to enroll near this threshold (i.e., no more than 15-16 participants), with a goal of including approximately half AWWE and half caregivers.

### Ethical approval

The University of Pittsburgh Institutional Review Board approved this study.

### Recruitment

We recruited participants from listservs of the Epilepsy Foundation and the Epilepsy Association of Western and Central Pennsylvania, a research registry from the University of Pittsburgh, and clinics in pediatric and adult neurology in western Pennsylvania. We included AWWE aged 14-26 years and caregivers to this population. We designated the minimum age as 14 years because, in the United States, 90% of females have undergone menarche by this age.^15^ We designated the maximum age as 26 years because our children’s hospital treats patients up to this age. This threshold aligns with the Society for Adolescent Health and Medicine’s definition of young adulthood as extending to 25 years.^16^ Our upper limit also matches data from the Centers for Disease Control and Prevention that defines the mean age at first birth in the United States as 27 years, representing a transition time in the reproductive years.^17^

We required participants to be able to read and write in English and to be able to access the internet. Although participants were not explicitly excluded due to intellectual or neurodevelopmental disability, the inclusion requirements for literacy and ability to interact with an internet-capable device resulted in the participants essentially being of typical intellectual development or with only mild intellectual or neurodevelopmental disabilities.

The study team screened potential participants for eligibility, reviewed a consent script, obtained verbal informed consent from individuals older than 18 years, and obtained consent from a guardian and assent from minors. The study team collected demographics at enrollment and consent. We compensated participants with a gift card for completing each study stage.

We invited participants who completed the first stage of the study (Brainstorming) to participate in the second stage (Sorting and Rating), although we also continued to recruit some additional participants for Sorting and Rating to account for attrition. For the Interpretation Meeting, we only invited participants who had completed the Sorting and Rating activities.

### Stage 1: Brainstorming

We opened the brainstorming activity from November 2023 through July 2024. Participants used Groupwisdom software to engage in this activity independently, asynchronously, and anonymously. Using free text entry, participants answered the prompt: “What should neurologists tell females with epilepsy (ages 14-26 years) about their reproductive health? List every important topic. Please list topics one-by-one.” Participants could see all previous responses from other participants. Two study team members each reviewed the initial list of items for duplicates and collaboratively decided which items to consolidate. A third study team member who is a young adult female with epilepsy reviewed the collated list compared with the original to ensure fidelity.

### Stage 2: Sorting and Rating

We opened sorting and rating activities from July through October 2024. Using Groupwisdom software, participants completed these activities independently, asynchronously, and anonymously. They could not see other participants’ responses.

In sorting, participants were instructed to “Group the statements on how similar in meaning they are to one another.” Participants collated the topics into thematically related categories and named each category.

In rating, participants rated each topic on two five-point Likert scales. The first was “How important is it for neurologists to tell adolescent females with epilepsy (ages 14-17 years old) about this topic? Please rate your answer on a scale from 1-very unimportant to 5-very important. Please try to use the full rating scale.” The second was “How important is it for neurologists to tell young adult females with epilepsy (ages 18-26 years old) about this topic? Please rate your answer on a scale from 1-very unimportant to 5-very important. Please try to use the full rating scale.”

### Data analysis

We created spatial point maps by applying multidimensional scaling to the sorting data. Each sorted topic is represented by a point on the map. The distance between any two points represents how often the corresponding topics were sorted together, reflecting their degree of thematic similarity. We performed hierarchical regression to define thematically related groups of items, which we refer to as “clusters.” This technique generates multiple candidate cluster solutions. In our study, the investigators selected the cluster solution that appeared to best model the data.

We used rating data to create a pattern matching display to visually compare clusters’ importance ratings for people with epilepsy aged 14-17 versus 18-26 years. To evaluate rating data for individual topics rather than clusters, we created a bivariate scatterplot demonstrating importance ratings for ages 14-17 versus 18-26 years. We calculated Pearson correlation coefficients for importance ratings for ages 14-17 versus 18-26 years, ages 14-17 years as rated by AWWE versus caregivers, and ages 18-26 years as rated by AWWE versus caregivers.

### Stage 3: Interpretation Meeting

We invited participants who completed Sorting and Rating to register to attend an interpretation meeting in October 2024, capping the total participants around 15 to ensure a manageable discussion. We held the meeting on Zoom teleconference software, and it lasted one hour. We did not require participants to share their name with other participants or appear on camera. The study team asked structured questions, aiming to achieve consensus on key topics. The study team took detailed field notes during the meeting, including transcription of verbal statements and capture of comments included in Zoom’s “Chat” function.

## Results

Fifty-four individuals participated in brainstorming, including 34 AWWE and 20 caregivers. Fifty-eight participants engaged in sorting and rating, including 35 AWWE and 23 caregivers. Sixteen individuals attended the participatory interpretation meeting, including seven AWWE and nine caregivers. Demographic characteristics are described in Table 1.

### Brainstorming

Participants generated 179 items during brainstorming. After the study team removed duplicates, there were 37 distinct counseling topics. Examples of topics include “whether epilepsy or seizure medications impact periods (menstrual cycles)” and “whether or not epilepsy is hereditary (whether or not it can be passed onto children).” A full list of topic statements is available in Table 2.

### Sorting

**FIGURE 2.**
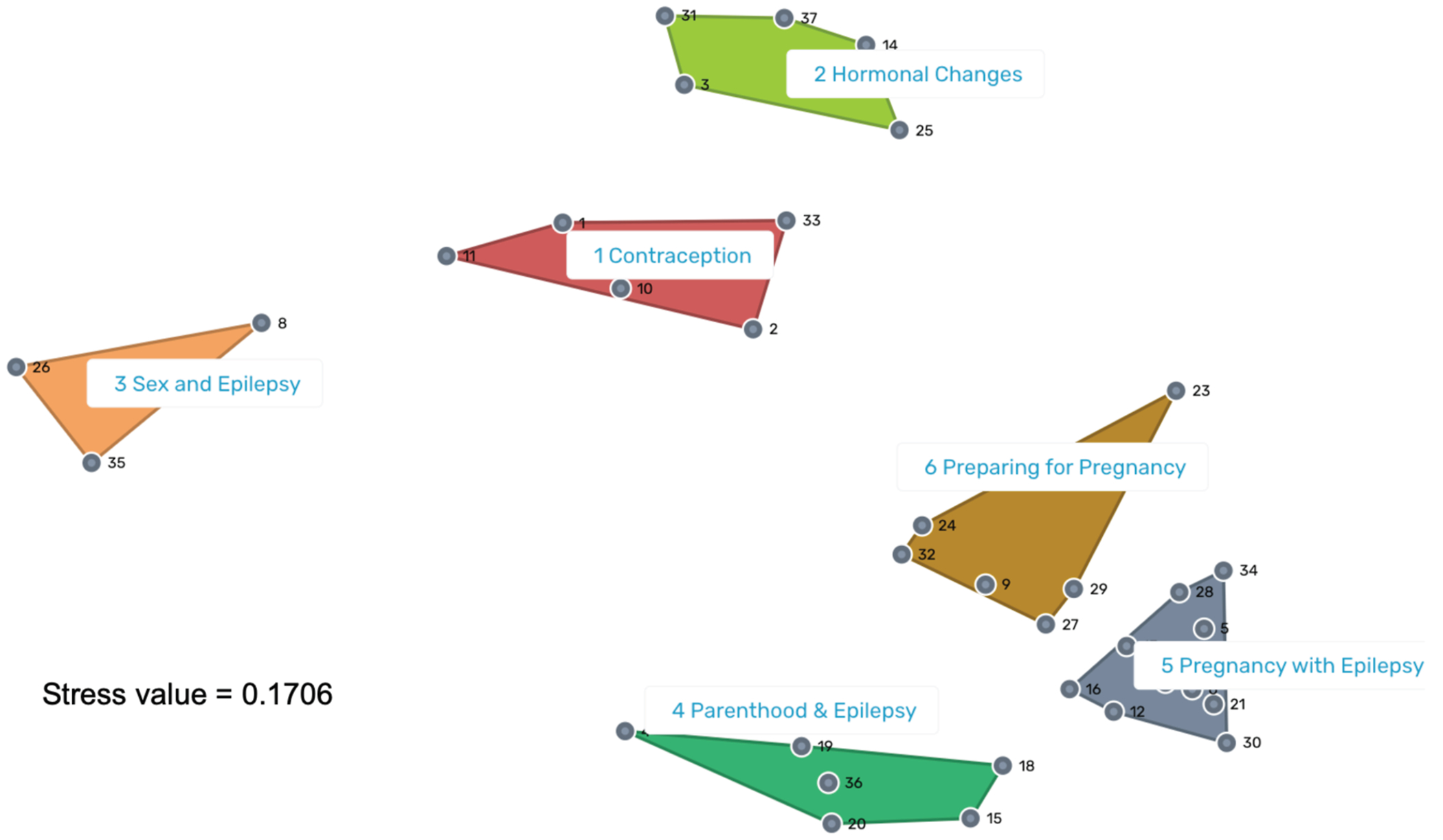
Cluster map. The color version of this figure is available in the online edition.

Figure 2 displays the cluster map overlaying the spatial point map. The stress value for our spatial point map was 0.17. A stress value is a measure of how well the spatial point map fits the sorting data, with lower values indicating better fit.^9,18^ Most stress values range from 0.20 to 0.36 with a mean of 0.28.^9,18^ We selected a six-cluster solution as the best fit for the data. We assigned preliminary names to clusters based on candidate names provided by participants, with final names pending the interpretation meeting. In the interpretation meeting, participants approved the preliminary names, which were “Contraception,” “Hormonal Changes,” “Sex & Epilepsy,” “Parenthood & Epilepsy,” “Pregnancy with Epilepsy,” and “Preparing for Pregnancy.”

Example items in “Contraception” include “contraceptive (birth control) counseling, including types of contraceptives available and their risks and benefits” and “whether or not using contraception (birth control) can cause seizures or affect epilepsy.” Example items in “Hormonal Changes” include “the relationship between hormone changes during puberty and epilepsy (i.e., whether changing hormones during puberty can cause epilepsy or increase seizures)” and “whether or not epilepsy and antiseizure medications cause abnormalities in hormone levels.” Examples in “Sex & Epilepsy” include “how epilepsy and antiseizure medications affect sexual function” and “importance of seizure first aid training for partners.” Examples in “Parenthood & Epilepsy” include “whether or not it is safe to breastfeed while taking antiseizure medications” and “whether antiseizure medications and doses need to change in the post-partum period.” Examples in “Pregnancy with Epilepsy” include “how epilepsy impacts pregnancy, including whether seizures harm the fetus (baby)” and “whether taking antiseizure medications causes problems carrying a pregnancy to full-term (i.e., increased risk of miscarriages or preterm/premature births).” Examples in “Preparing for Pregnancy” include “whether and how epilepsy and antiseizure medications impact fertility and the ability to become pregnant” and “the importance of planning pregnancies and avoiding unplanned pregnancies.”

### Cluster ratings

We used the importance ratings participants assigned to each topic to generate cluster ratings on a five-point Likert scale. Cluster ratings for importance for ages 14-17 years ranged from 3.63 for “Parenthood & Epilepsy” to 4.50 for “Contraception.” For ages 14-17 years, two clusters did not achieve a rating of at least 4.00: “Parenthood & Epilepsy” and “Pregnancy with Epilepsy.”

**FIGURE 3.**
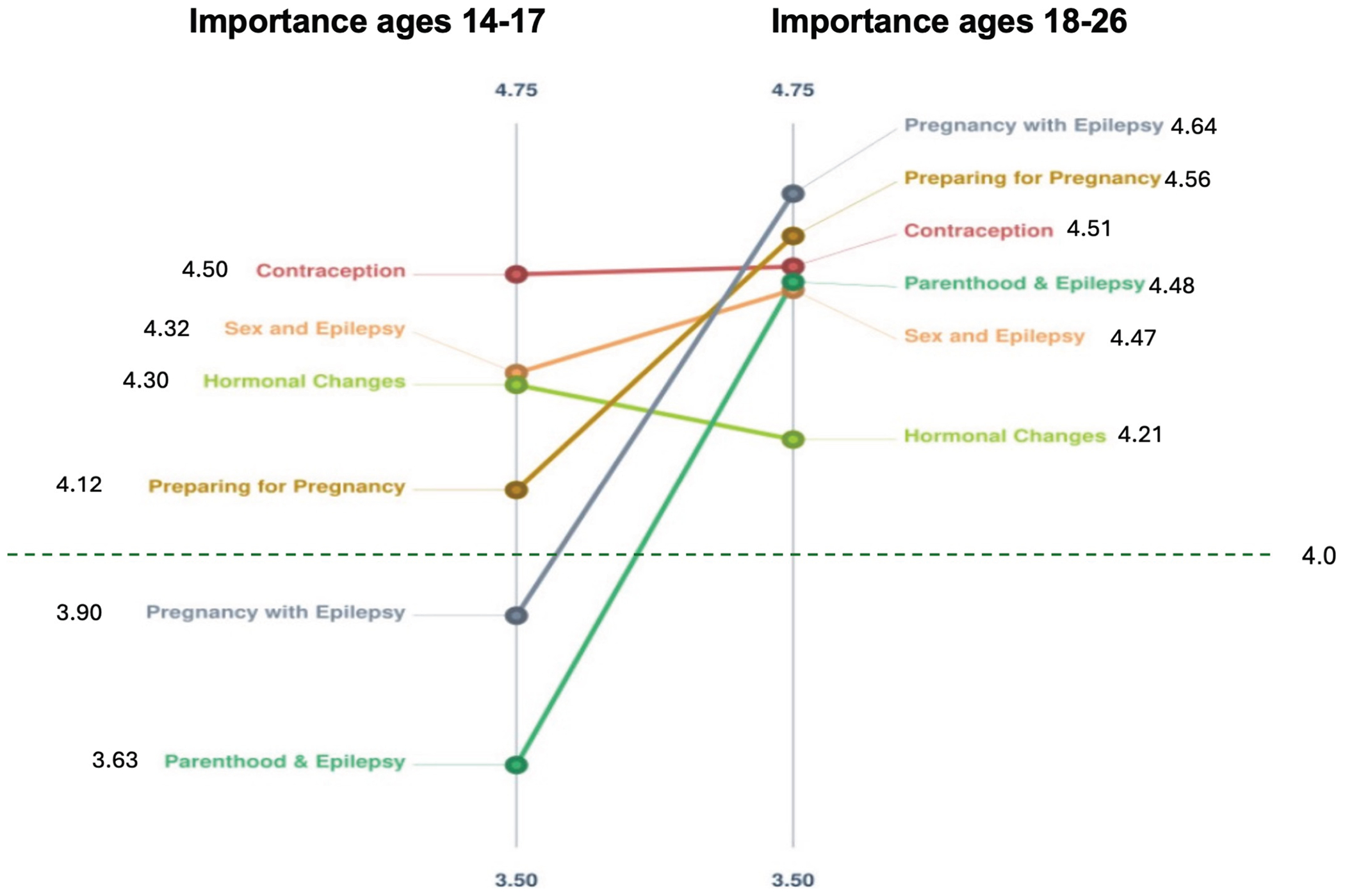
Pattern matching display for importance ratings of clusters. The color version of this figure is available in the online edition.

Cluster ratings for importance for 18-26 years ranged from 4.21/5.00 for “Hormonal Changes” to 4.64 for “Pregnancy with Epilepsy.” All clusters achieved a rating of at least 4.00. Five of six clusters were rated higher in importance for ages 18-26 years compared with ages 14-17 years. The sole exception, “Hormonal Changes,” was rated 4.21 for ages 18-26 years compared with 4.30 for ages 14-17 years. Cluster ratings for ages 14-17 versus 18-26 years are depicted in Fig 3.

### Individual topic ratings

**FIGURE 4.**
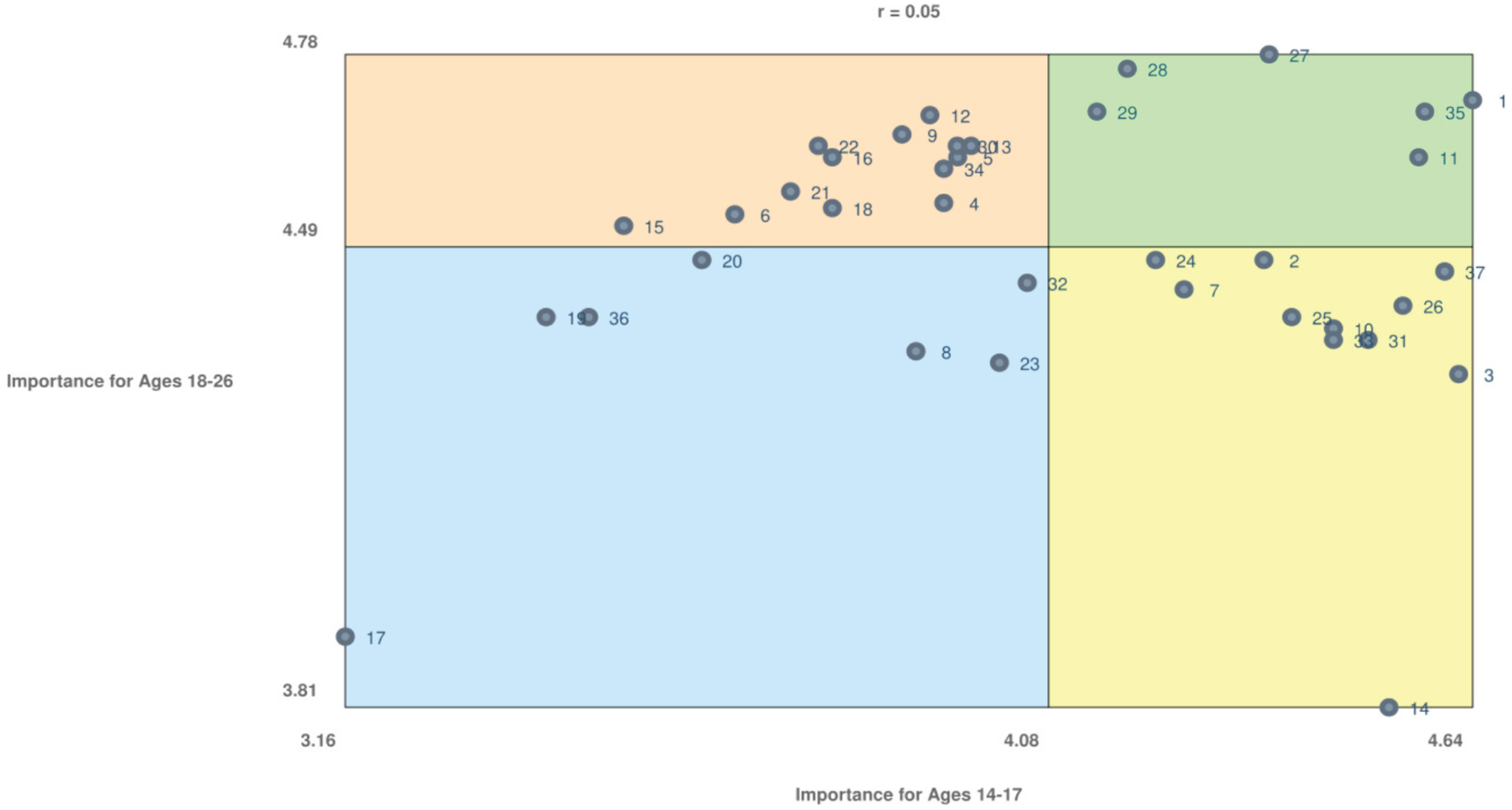
Plot of importance for ages 14-17 versus ages 18-26. The color version of this figure is available in the online edition.

Figure 4 displays a bivariate scatterplot of individual topic ratings for ages 14-17 versus 18-26 years. The plot is divided into quadrants, with the upper right green quadrant representing higher importance for both age groups and the lower left blue quadrant representing lower importance for both age groups.

Six topics rated with high importance for both age groups included “drug interactions between antiseizure medications and contraceptives (birth control),” “importance of seizure first aid training for partners,” “whether or not using contraception (birth control) can cause seizures or affect epilepsy,” “the importance of letting your doctor know if you are pregnant or trying to become pregnant,” “whether or not antiseizure medications should be stopped or changed if someone becomes pregnant,” and “whether or not pregnancy is safe for people with epilepsy.”

Seven topics rated with lower importance for both age groups included “how hormonal changes during menopause can affect epilepsy and seizure control,” “whether antiseizure medications and doses need to change in the post-partum period,” “whether or not epilepsy increases the risk of post-partum depression,” “postpartum concerns such as managing seizures while caring for a newborn,” “how epilepsy and antiseizure medications affect sexual function,” “whether antiseizure medications can affect pregnancy test results (false positive or false negative),” and the “importance of taking daily folic acid supplementation.”

Participants rated 11 items relatively higher in importance for ages 14-17 years and lower in importance for ages 18-26 years; the item rated lowest for ages 18-26 years (out of all items) was the “relationship between hormone changes in puberty and epilepsy (i.e., whether changing hormones during puberty can cause epilepsy or increase seizures).” Participants rated 13 items relatively higher in importance for ages 18 to 26 years and lower in importance for ages 14 to 17 years, most of which pertained to pregnancy and parenthood. Individual item ratings are detailed in Table 3.

The Pearson correlation coefficient of importance ratings between ages 14-17 and 18-26 years was r = 0.05, indicating a very weak positive correlation.

### Ratings of AWWE versus caregivers

For importance ratings for ages 14-17 years, the Pearson correlation coefficient for ratings between AWWE and caregivers was 0.9, indicating a strong positive correlation. For importance ratings for ages 18-26 years, the Pearson correlation coefficient for ratings between AWWE and caregivers was 0.76, indicating a moderately strong positive correlation.

### Interpretation meeting

Participants believed that the clusters reflected the important topics for counseling (according to two participants: “I think all these clusters are relevant” and “these were the important questions I had”). Participants agreed that topics should vary by age group (14-17 versus 18-26 years) and felt that our findings were age-appropriate (as one participant said, the topics were “well-selected for age groups,” and another noted, “those ratings seem pretty spot-on for each demographic.”)

Compared with the 2017 AAN quality measure, participants preferred the counseling topics suggested by our findings due to greater perceived thoroughness (“Your topics are more comprehensive”), patient centeredness (“Your topic list is more patient-friendly, detailed, and applicable”), and age appropriateness (“Your topic list is more age-appropriate.”)

Participants reflected on why folic acid supplementation was rated of lower importance across age groups despite being prioritized in the AAN quality measure. Participants revealed that they had either not been told about folic acid supplementation (“It’s not a topic that’s ever been discussed at any neuro appointment”) or they had been told to take daily folic acid supplements without a clear rationale (“Folic acid was prescribed but no one explained why”). After the study team shared information about folic acid supplementation, participants wanted to recategorize this topic as important for all ages (“Now I’d put it in the most important for everyone category”).

Participants struggled to achieve consensus when reflecting on the ranking of the teratogenic and neurodevelopmental effects of ASMs in pregnancy as relatively lower in importance for the 14-17 age group (whereas the AAN guideline prioritizes this information for all patients older than 12 years). Some agreed that these topics are less important for ages 14-17 years (“I don’t think the topics would be of interest to that age group”), others felt it is important (“I do think it is important for this age category”), and still others expressed that counseling should be customized to the individual person (“I guess each patient’s experience is different and we need to meet the patient where they are at”).

Participants offered suggestions about how topics could be shared in neurology outpatient visits. A few participants suggested and many endorsed using a previsit checklist wherein AWWE and families could select topics to prioritize during the visit (“Could there be a checklist patients could fill out before going to the doctor that covers all topics and patients can choose what they’d like to discuss?”). Some participants also felt that such a tool could help prevent perhaps uncomfortable or unwanted conversations. Another idea was a “hotline or chatline” wherein AWWE and families could direct questions following visits. Some participants brought up supplementary written materials as helpful (“There’s power behind pamphlets and brochures”), but others expressed concern that AWWE and families might not read written materials and that written materials might be embarrassing if peers or other family members found them.

## Discussion

**FIGURE 5.**
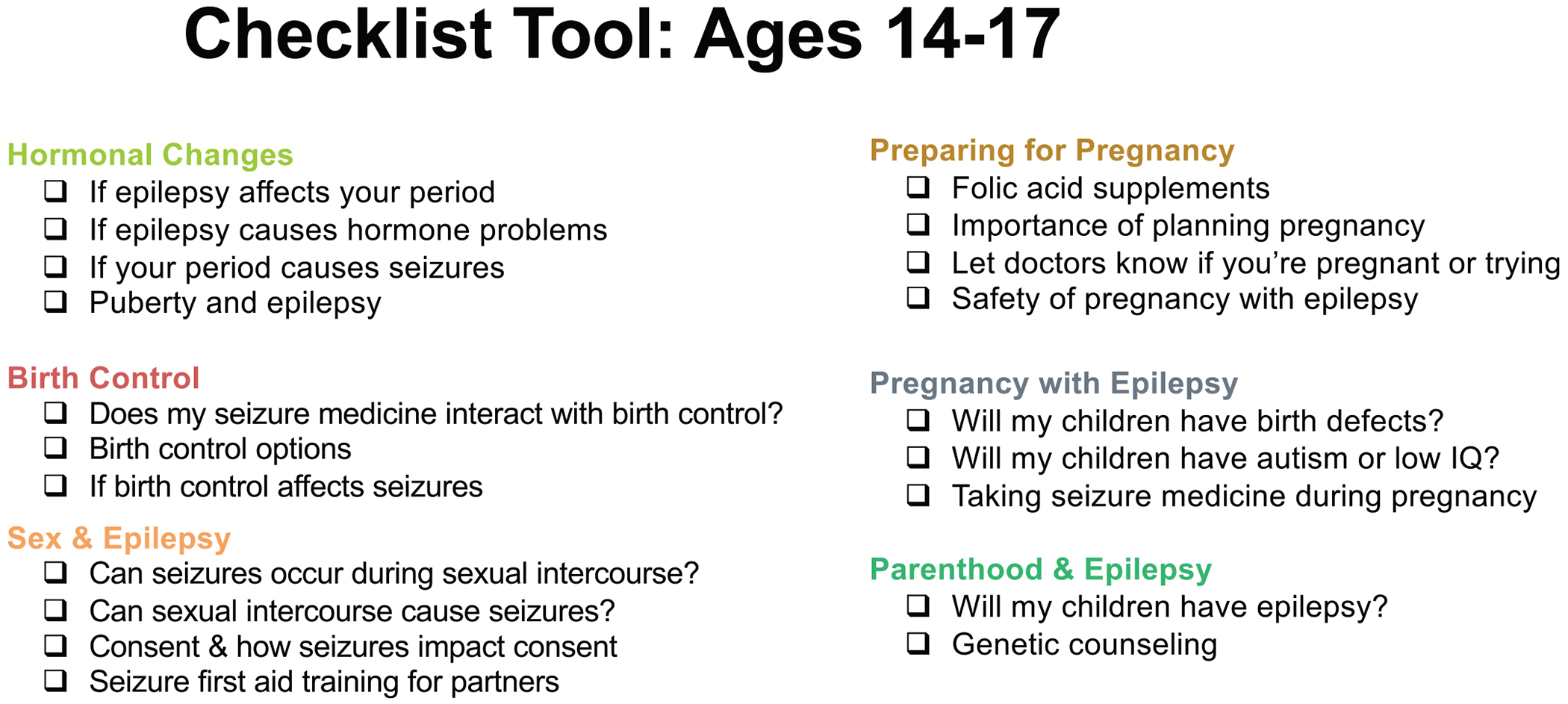
Checklist tool: ages 14-17.

**FIGURE 6.**
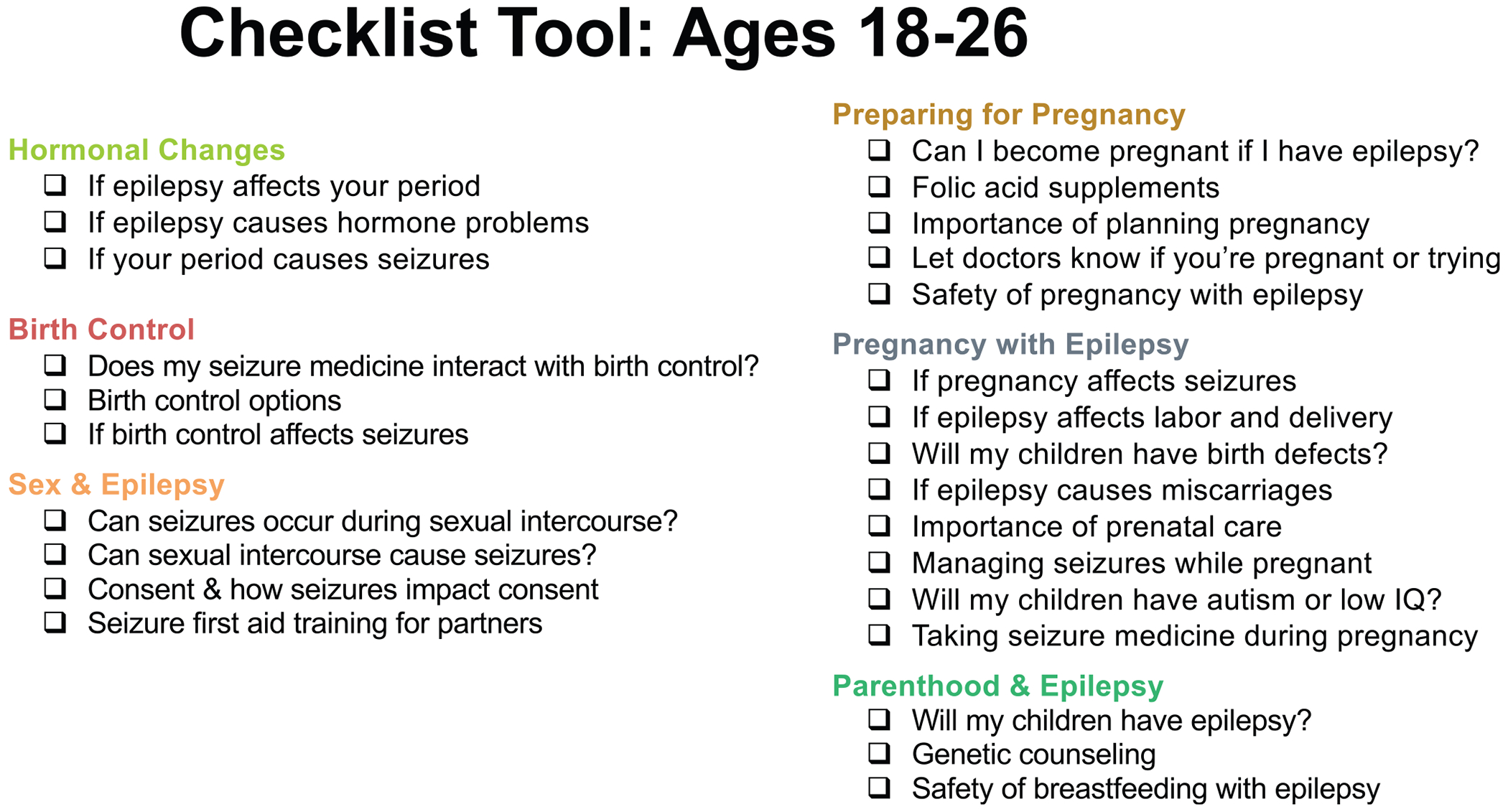
Checklist tool: ages 18-26.

Our study reveals the priorities and preferences of AWWE and their caregivers for reproductive health counseling in the neurology context. Participants were interested in a wider variety of counseling topics than those recommended in the current 2017 AAN quality measure.^8^ Participants also recommended that counseling topics be tailored by age group rather than being “one size fits all” for adolescents and adults as per current guidelines. AWWE and caregivers were closely aligned on their prioritization of counseling topics. Given the group’s support for a previsit checklist to personalize counseling, our team developed prototype checklists for AWWE ages 14-17 and 18-26 years based on the research findings, which are displayed in Figs 5 and 6.

In a recent survey, adult women with epilepsy also expressed a broader range of concerns about reproductive health and epilepsy than is currently recommended for routine counseling in the AAN guideline.^8,19^ The findings of this study were used by the Epilepsy and Pregnancy Medical Consortium, in partnership with the One8 Foundation to develop epilepsypregnancy.com, a comprehensive web site about reproductive health and epilepsy.^20^ Given that this study concerned only adults and primarily those who were considering or who had recently experienced pregnancy, the findings of this study cannot be generalized to our study population and its associated web site is also not tailored to our study population.^19,20^ However, this study also attests to the value of understanding the counseling and care preferences of women with epilepsy regarding reproductive health and epilepsy, and incorporating these preferences in clinical and educational tools.^19,20^

Our study adds to the growing literature on incorporating the viewpoints of people with lived experience with health conditions in developing clinical best practices and tools. Despite contemporary increasing interest in incorporating such perspectives,^21–23^ a recent scoping review identified few studies eliciting patient preferences in guideline development as well as a lack of clarity on optimal methodology for doing so.^23^ Concept Mapping has recently been used in several studies to incorporate the perspectives of people with lived experience when developing clinical implementation strategies, processes and procedures, and tools.^24–27^ Our study attests to the feasibility of Concept Mapping as one method for eliciting patient and caregiver preferences when developing clinical best practice guidelines and tools.

Our study findings can be incorporated into development of more patient-centered quality metrics and tools for counseling of AWWE. One strategy, as suggested by our participants, may be use of a previsit checklist wherein AWWE and caregivers can indicate high-priority topics. The intention is for the tool to be a convenient means for patients/families to communicate their preferences for in-visit counseling, which clinicians would consider when agenda setting for the visit in conjunction with clinician-prioritized topics. Such a tool may increase time efficiency of this counseling, which is important given that pediatric neurologists have cited limited time as a key barrier to performing this counseling.^28,29^ Previsit checklist tools for agenda setting have been studied in other settings, including primary care and oncology.^30–32^

Our research team developed prototypes of checklists that can be used for eliciting patient/family preferences for counseling about reproductive health and epilepsy. These prototypes can be refined in partnership with AWWE and caregivers to ensure usability, including by considering clarity of language and accessibility of presentation as well as testing feasibility of using the checklists in clinical practice. Our research team is also developing a training for pediatric neurologists on counseling AWWE about reproductive health and epilepsy, and this training could be a dissemination mechanism for this counseling approach. We also recommend potentially expanding the checklist beyond reproductive health to cover other important counseling areas such as safety precautions, driving, sudden unexpected death in epilepsy, mental health, and concerns specific to employment and educational settings.

However, our findings also attest to the importance of incorporating clinician-driven priorities into counseling agendas alongside patient/family priority topics. For example, although folic acid supplementation is a priority topic in the AAN quality measure, participants in our study rated folic acid supplementation poorly initially.^8^ Yet, participants explained in the interpretation meeting that they had never received clear information about folic acid, and after receiving such information from the study team, they wanted folic acid supplementation rated as a highly important topic for counseling across all age groups. Therefore, agenda setting must be a delicate balance between what clinicians and patients/families deem most important to discuss.

There are several limitations of the study. Our study focuses on the United States, and thus findings may not be generalizable to other nations. Furthermore, our study oversampled western Pennsylvania, which may limit generalizability to other parts of the country. The oversampling of western Pennsylvania resulted in strengths and limitations of the sample that reflect this region. For example, given that many people in western Pennsylvania live in rural areas, we were able to oversample people who live in rural areas, which is important given that this population is at higher risk of adverse reproductive health outcomes compared with people in urban areas.^33–36^ However, by contrast, western Pennsylvania has limited racial and ethnic diversity, and our sample includes a lower percentage of people identifying as black and/or Hispanic/Latino compared with the general US population.^37,38^ Furthermore, due to the inability of offering the Concept Mapping software in translation, we only included English-speaking participants, which also limits generalizability. The caregiver sample reported relatively high education levels, which is also a limitation. Future directions include validation of the research findings with a sample that is more diverse in terms of race, ethnicity, primary language, and educational attainment. Another future direction is further research to ascertain the counseling priorities of AWWE diagnosed with intellectual disability and their caregivers.

## Conclusion

AWWE and their caregivers desire comprehensive counseling about reproductive health and epilepsy that is tailored to age group and customizable to individual AWWE and families. The preferences and priorities of AWWE and caregivers should be incorporated in future guideline and clinical best practice development. Our study attests to the value and feasibility of Concept Mapping as a method to ascertain the preferences of AWWE and caregivers for integration into clinical best practice guidelines and tools. Future studies should explore the impact of personalized approaches to reproductive health counseling for AWWE and caregivers, including on satisfaction with care and health outcomes.

## Figures and Tables

**TABLE 1.** Participant Demographics

Demographic Variable	AWWE (n = 45)	Caregivers (n = 30)
Median age (IQR)	19 (17-23)	47 (43-52.5)
Region type		
Rural	12 (27%)	8 (26%)
Suburban	22 (49%)	17 (57%)
Urban	11 (24%)	5 (17%)
Race		
Asian	1 (2%)	2 (7%)
Black	4 (9%)	1 (3%)
Multiracial	4 (9%)	0 (0%)
Native American	1 (2%)	0 (0%)
White	35 (78%)	27 (90%)
Ethnicity		
Hispanic/Latino	6 (13%)	3 (10%)
Education level		
Middle school or less	8 (18%)	1 (5%)
Some high school	1 (2%)	0 (0%)
High school diploma or GED	10 (22%)	1 (5%)
Some college no degree	12 (27%)	3 (14%)
Associate’s degree	1 (2%)	(5%)
Bachelor’s degree	6 (13%)	7 (24%)
Master’s degree	1 (2%)	7 (33%)
Professional or doctoral degree	0 (0%)	3 (14%)
Declined to respond	6 (14%)	7
Region		
Census division		
Northeast	38 (85%)	28 (93%)
Midwest	3 (7%)	2 (7%)
West	2 (4%)	0 (0%)
South	2 (4%)	0 (0%)
Pennsylvania	33 (73%)	25 (83%)

Abbreviations:

AWWE = Adolescent and young adult women with epilepsy

GED = General Educational Development

IQR = Interquartile range

**TABLE 2.** Full List of Statements Included in Sorting and Rating

Item Number	Statement
1	Drug interactions between antiseizure medications and contraceptives (birth control)
2	Referrals to gynecologists or other reproductive health providers for reproductive health care
3	Whether epilepsy or seizure medications impact periods (menstrual cycle)
4	Whether or not epilepsy is hereditary (whether or not it can be passed onto children)
5	How pregnancy impacts epilepsy and seizures (i.e., is there an increased risk of seizures in pregnancy?)
6	How epilepsy affects labor and delivery
7	The relationship between epilepsy/antiseizure medication and sexual intercourse (i.e., Can you have a seizure during sexual intercourse? Can sexual intercourse cause seizures?)
8	How epilepsy and antiseizure medications affect sexual function
9	Whether and how epilepsy and antiseizure medications impact fertility and the ability to become pregnant
10	Contraceptive (birth control) counseling, including types of contraceptives available and their risks and benefits
11	Whether or not using contraception (birth control) can cause seizures or affect epilepsy
12	How epilepsy impacts pregnancy, including whether seizures harm the fetus (baby)
13	Whether taking antiseizure medications in pregnancy increases the risk of birth defects
14	Relationship between hormone changes during puberty and epilepsy (i.e., whether changing hormones during puberty can cause epilepsy or increase seizures)
15	Whether or not it is safe to breastfeed while taking antiseizure medication
16	Whether taking antiseizure medicines causes problems carrying a pregnancy to full-term (i.e., increase risk of miscarriages or preterm/premature births)
17	How hormonal changes during menopause can affect epilepsy and seizure control
18	Offer genetic counseling if there are concerns about passing epilepsy or related conditions to children
19	Whether antiseizure medications and doses need to change in the post-partum period
20	Post-partum concerns such as managing seizures while caring for a newborn
21	Discuss the importance of regular prenatal care during pregnancy, including monitoring for complications related to epilepsy and medications
22	How to manage having seizures during pregnancy as safely as possible
23	Importance of taking daily folic acid supplementation
24	Importance of planning pregnancies and avoiding unplanned pregnancies
25	Whether or not epilepsy and antiseizure medications cause abnormalities in hormone levels
26	Importance of consent for sexual intercourse, including how having seizures impacts ability to provide consent
27	The importance of letting your doctor know if you are pregnant or trying to become pregnant
28	Whether or not antiseizure medications should be stopped or changed if someone with epilepsy becomes pregnant
29	Whether or not pregnancy is safe for people with epilepsy
30	Whether there are long-term neurodevelopmental effects on the fetus (baby) with the use of antiseizure medications during pregnancy
31	Importance of keeping a monthly calendar tracking seizures for a pattern related to the menstrual cycle
32	Whether antiseizure medication can affect pregnancy test results (false positive or false negative)
33	Importance of informing people with epilepsy that doctors give better advice about reproductive health than Google
34	Why taking antiseizure mediations as prescribed is important in pregnancy
35	Importance of seizure first aid training for partners
36	Whether or not epilepsy increases the risk for postpartum depression
37	Whether and how menstruation (periods) and the menstrual cycle affect seizures and epilepsy

Item numbers are included to facilitate interpretation of Figs 2 and 4.

**TABLE 3.** Individual Items With Clusters and Importance Ratings

Item	Cluster	Importance: Ages 14-17	Importance: Ages 18-27
Whether using birth control can cause seizures or affect epilepsy	Contraception	4.57	4.63
Drug interactions between antiseizure medications and birth control	Contraception	4.64	4.71
Importance of informing people with epilepsy that doctors give better advice about reproductive health than Google	Contraception	4.46	4.36
Referrals to gynecologists or other reproductive health providers for reproductive health care	Contraception	4.36	4.47
Birth control counseling, including types of contraceptives available and their risks and benefits	Contraception	4.45	4.37
How hormonal changes during menopause can affect epilepsy and seizure control	Hormone changes	3.16	3.92
Whether or not epilepsy and antiseizure medications cause abnormalities in hormone levels	Hormone changes	4.40	4.39
Whether epilepsy or seizure medications impact periods (menstrual cycles)	Hormone changes	4.62	4.31
Importance of keeping a monthly calendar tracking seizures for a pattern related to the menstrual cycle	Hormone changes	4.50	4.36
Whether and how menstruation (periods) and the menstrual cycle affect seizures and epilepsy	Hormone changes	4.60	4.46
Relationship between hormone changes during puberty and epilepsy (i.e., whether changing hormones during puberty can cause epilepsy or increase seizures)	Hormone changes	4.53	3.81
The relationship between epilepsy/antiseizure medication and sexual intercourse (i.e., Can you have a seizure during sexual intercourse? Can sexual intercourse cause seizures?)	Sex & epilepsy	4.26	4.43
How epilepsy and antiseizure medications affect sexual function	Sex & epilepsy	3.91	4.34
Importance of seizure first aid training for partners	Sex & epilepsy	4.57	4.69
Importance of consent for sexual intercourse, including how having seizures impact ability to provide consent	Sex & epilepsy	4.55	4.41
Whether or not epilepsy is hereditary (whether or not it can be passed onto children)	Parenthood & epilepsy	3.95	4.56
Whether antiseizure medications and doses need to change in the post-partum period	Parenthood & epilepsy	3.42	4.39
Offer genetic counseling if there are concerns about passing epilepsy or related conditions to children	Parenthood & epilepsy	3.80	4.55
Whether or not epilepsy increases the risk of post-partum depression	Parenthood & epilepsy	3.48	4.39
Whether or not it is safe to breastfeed while taking antiseizure medication	Parenthood & epilepsy	3.53	4.53
Post-partum concerns such as managing seizures while caring for a newborn	Parenthood & epilepsy	3.63	4.47
Why taking antiseizure medications as prescribed is important in pregnancy	Pregnancy with epilepsy	3.95	4.61
Whether taking antiseizure medications in pregnancy increases the risk of birth defects	Pregnancy with epilepsy	3.98	4.64
Whether taking antiseizure medications causes problems carrying a pregnancy to full-term (i.e., increased risk of miscarriages or preterm/premature births)	Pregnancy with epilepsy	3.80	4.63
How epilepsy impacts pregnancy, including whether seizures harm the fetus (baby)	Pregnancy with epilepsy	3.92	4.69
Whether there are long-term neurodevelopmental effects on the fetus (baby) with the use of antiseizure medications during pregnancy	Pregnancy with epilepsy	3.96	4.64
Whether or not antiseizure medications should be stopped or changed if someone with epilepsy becomes pregnant	Pregnancy with epilepsy	4.19	4.76
How pregnancy impacts epilepsy and seizures (i.e., is there an increased risk of seizures in pregnancy?)	Pregnancy with epilepsy	3.96	4.63
How to manage having seizures during pregnancy as safely as possible	Pregnancy with epilepsy	3.78	4.64
How epilepsy affects labor and delivery	Pregnancy with epilepsy	3.67	4.54
The importance of regular prenatal care during pregnancy, including monitoring for complications related to epilepsy and medications	Pregnancy with epilepsy	3.75	4.58
Importance of taking daily folic acid supplementation	Preparing for pregnancy	4.02	4.32
Whether or not pregnancy is safe for people with epilepsy	Preparing for pregnancy	4.15	4.69
The importance of letting your doctor know if you are pregnant or trying to become pregnant	Preparing for pregnancy	4.37	4.78
Whether and how epilepsy and antiseizure medications impact fertility and the ability to become pregnant	Preparing for pregnancy	3.89	4.66
Whether antiseizure medication can affect pregnancy test results (false positive or false negative)	Preparing for pregnancy	4.05	4.44
Importance of planning pregnancies and avoiding unplanned pregnancies	Preparing for pregnancy	4.22	4.47
